# Numerical analysis of vibration modes of a qPlus sensor with a long tip

**DOI:** 10.3762/bjnano.12.7

**Published:** 2021-01-21

**Authors:** Kebei Chen, Zhenghui Liu, Yuchen Xie, Chunyu Zhang, Gengzhao Xu, Wentao Song, Ke Xu

**Affiliations:** 1 School of Nano Technology and Nano Bionics, University of Science and Technology of China, Suzhou 215123, China; 2 Platform for Characterization and Test, Suzhou Institute of Nano-Tech and Nano-Bionics, Chinese Academy of Sciences (CAS), Suzhou 215123, China; 3 CAS Key Laboratory of Nanophotonic Materials and Devices, Suzhou Institute of Nano-Tech and Nano-Bionics, Suzhou 215123, China

**Keywords:** finite element method, long tilted tip, noncontact atomic force microscopy, qPlus sensor, quartz tuning fork, simulations

## Abstract

We study the oscillatory behavior of qPlus sensors with a long tilted tip by means of finite element simulations. The vibration modes of a qPlus sensor with a long tip are quite different from those of a cantilever with a short tip. Flexural vibration of the tungsten tip will occur. The tip can no longer be considered as a rigid body that moves with the prong of the tuning fork. Instead, it oscillates both horizontally and vertically. The vibration characteristics of qPlus sensors with different tip sizes were studied. An optimized tip size was derived from obtained values of tip amplitude, ratio between vertical and lateral amplitude components, output current, and quality factor. For high spatial resolution the optimal diameter was found to be 0.1 mm.

## Introduction

Quartz tuning forks are widely used in the watch industry because of their low frequency offset over a wide temperature range [1]. In addition, quartz tuning forks have a high elastic constant, a high quality factor (*Q* factor), and are self-sensing due to the piezoelectric effect [1]. Therefore, a quartz tuning fork can be used as a force sensor. The central part of the “qPlus sensor” is a quartz tuning fork of which one prong is fixed onto a substrate and the other prong with an attached tip serves as a self-sensing cantilever [2]. In 1996, F. J. Giessibl et al. first used the qPlus sensor to measure the morphology of a grating and a CD at room temperature [3]. Since then, this technique has been used extensively in the fields of physics, chemistry, and materials science to obtain images of high spatial resolution [4–12].

Most research under low-temperature and ultrahigh-vacuum (LT-UHV) conditions has been carried out using the first-order eigenmode of the qPlus sensor, which has a short tip that can be considered as a rigid body vertically attached to the tuning fork prong. However, soft biological samples, such as living cells and lipid membranes [13–15], must be immersed in a liquid environment to maintain their original properties. In order to avoid immersion of the tuning fork and the wiring in the liquid, a longer tip is required. By keeping the nodes of the tip in the higher-order modes close to the liquid surface, the frequency drift of the sensor can be effectively limited, maintaining a high *Q* factor [16–17]. By using a qPlus sensor with a long tip, atomic resolution and near-field optical images were obtained in liquid environments [15,18–19].

**Figure 1:**
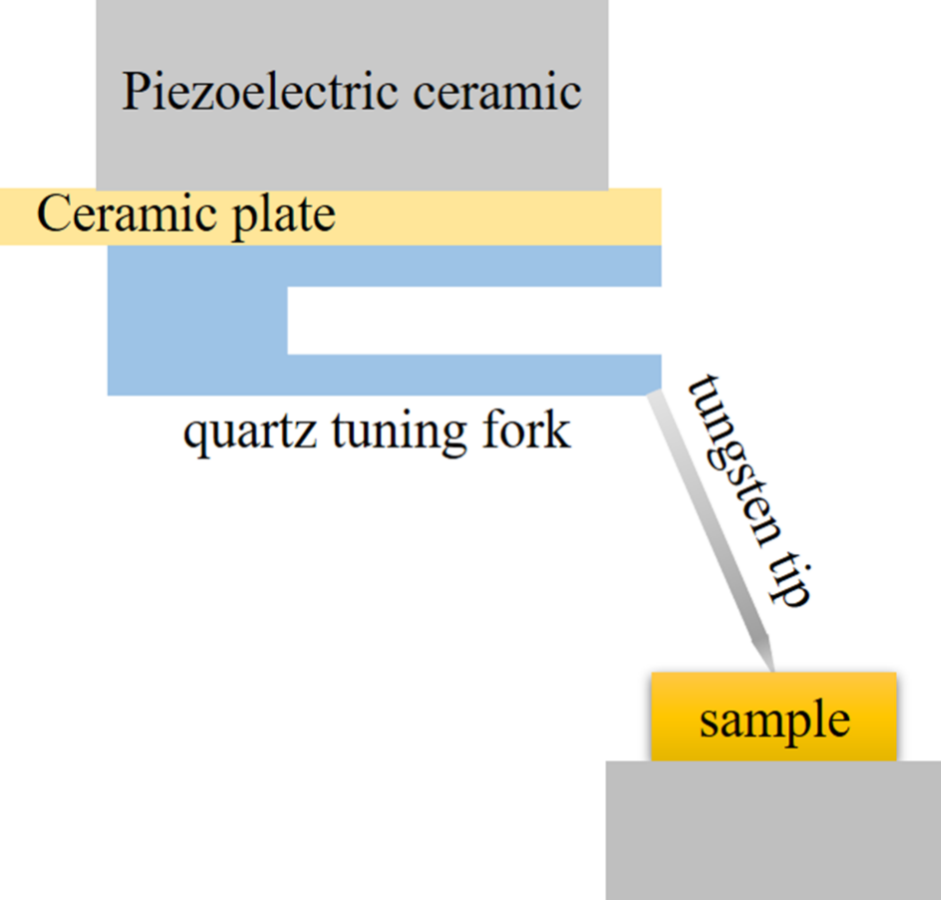
Schematic diagram of a qPlus sensor with a long tilted tip.

For some applications, it is required that the tip is not perpendicular to the prong of the tuning fork [20–21]. The advantage of a qPlus sensor with a long tilted tip (Figure 1) is that forces in multiple directions can be detected due to the multi-directional vibration of the tip [17]. Furthermore, by using a qPlus sensor with a long tilted tip, vertical incident light can be coupled to the tip apex. This setup has the added benefit of locating the exact target location with high resolution when it is combined with an optical microscope to observe the sample surface from upside [22–23]. However, there is only scarce work regarding the analysis of how the shape of the long tilted tip affects the vibration of the sensor. The selection of tip length and diameter often depends on the experience of researchers.

Here, we report a numerical study of the vibration modes of qPlus sensors. Eigenfrequencies, tip amplitudes, ratios between vertical and lateral amplitude components, output currents, and *Q* factor values as functions of the tip size of the qPlus sensor are systematically analyzed. Eventually, the optimal tip size can be derived from the simulation results.

## Methods

### Model and parameters

The model in the simulation is based on an MS1V-T1K-type quartz tuning fork used in our experiment (details will be described later). The dimensions of the MS1V-T1K quartz tuning fork, length *L* = 3423 µm, width *W* = 687 µm, and thickness τ = 121 µm, are used in the finite element model. The arrangement of the gold electrodes on the tuning fork is the same as in reality. One electrode pair is grounded and the other is set to a virtual ground. The calculated mechanical eigenfrequency of the bare tuning fork is 32.19 kHz. After considering the electrostatic and piezoelectric effects, the eigenfrequency is shifted to 32.23 kHz, which is only 0.12% higher. Therefore, the effect of the shunt capacitance of the tuning fork is negligible.

**Figure 2:**
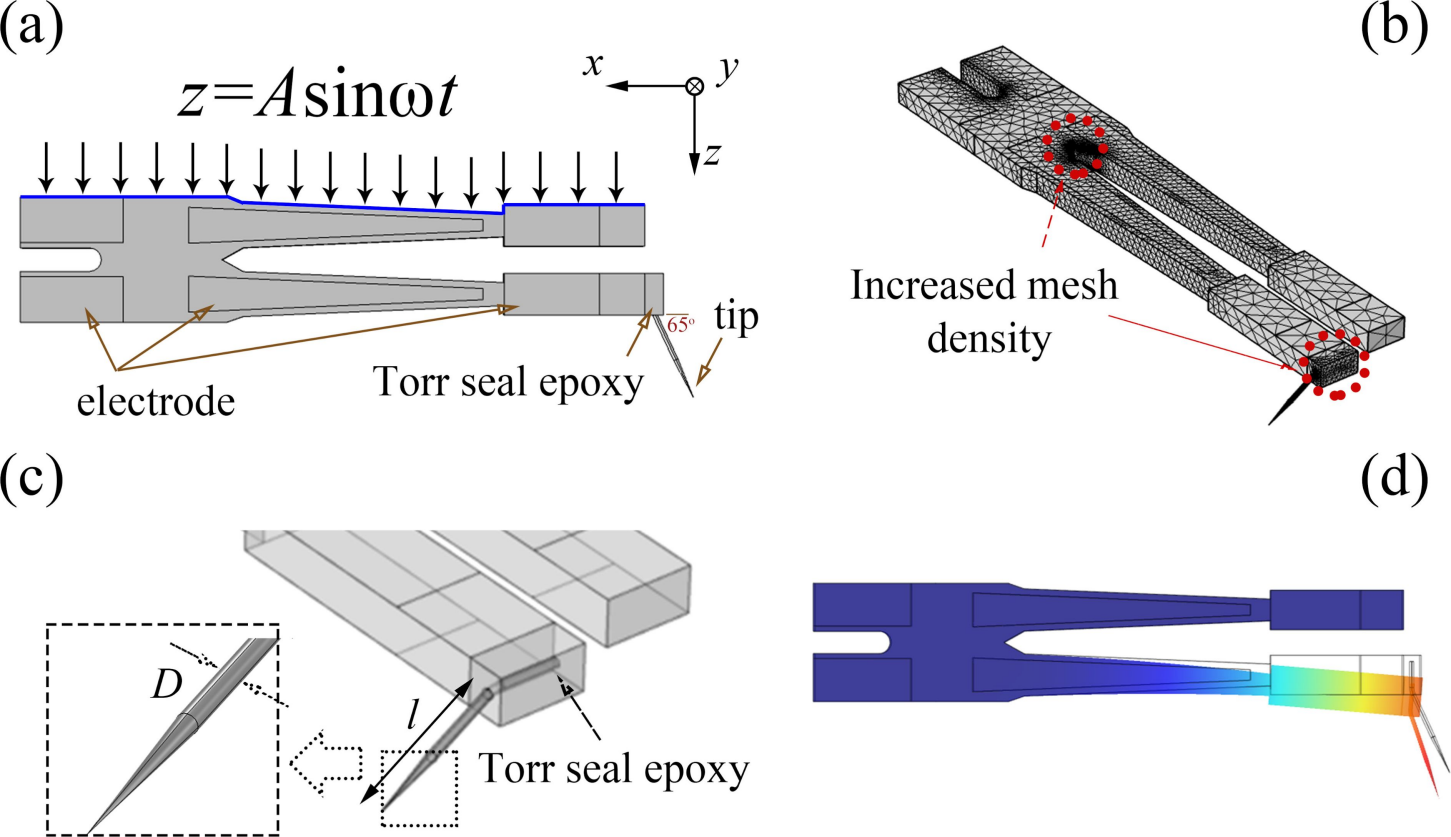
(a) Schematic diagram of the simulation model including tuning fork, Torr seal epoxy glue, and tungsten tip. Mechanical vibration excitation is applied on one side of the tuning fork. (b) Map of the mesh distribution as implemented in the finite element simulation. (c) Zoomed-in view of the tungsten wire attached to the end of the tuning fork. (d) False-color representation of the total displacement levels when the free prong deforms under excitation.

As shown in Figure 2a, the tungsten tip is attached to one prong using a rectangular drop of Torr seal epoxy. The diameter values of the tungsten tip used in the simulations were chosen according to diameters of the tungsten wire available [24]. We selected four different diameters: 0.025 mm, 0.05 mm, 0.075 mm, and 0.1 mm. The tip length can be customized according to the experimental needs. In this paper, nine lengths are selected for calculation at each diameter: 0.5 mm, 0.65 mm, 0.8 mm, 1.0 mm, 1.2 mm, 1.4 mm, 1.5 mm, 1.7 mm, and 2 mm. The angle between the tip and the prong is 65°. An external excitation is applied by exerting a harmonic displacement of 4.5 nm on the side of the tuning fork prong without a tip. Figure 2b visualizes the mesh distribution used for the calculations. The mesh density increases at the link between the two prongs and near the boundaries of each material. Figure 2c is a zoomed view of the tungsten wire attached to the end of the tuning fork. The epoxy glue is defined as a cuboid in order to facilitate mesh generation and reduce calculation time, since it is not guaranteed to have a fixed geometry after curing. The glue thickness in the simulation is set to be the average glue thickness of the qPlus sensor used in our experiment. In this case, the volume of the glue in the simulation is approximately equal to the volume of the glue in the experiment. Figure 2d is a false-color representation of the total displacement levels. Warmer colors represent a larger displacement.

All calculations were carried out using COMSOL Multiphysics. Table 1 summarizes the parameters used, including Young’s modulus, Poisson’s ratio, mass density, and damping coefficients for all materials considered. The values for Torr seal epoxy were chosen as in the papers by Dennis van Vörden et al. [25] and Omur E. Dagdeviren and co-workers [26]. The parameters for quartz, gold, and tungsten were taken from the materials library of the simulation software, except for the damping coefficient for quartz, which was chosen based on our experimental results. According to [26], it is also worth noting that (i) due to the comparatively low internal damping occurring inside gold and tungsten, we did not assign a damping coefficient to any of these materials to reduce the computational cost, and that (ii) the sensor is oscillating in vacuum.

**Table 1:** Material parameters used for finite element calculations.

Material constant	Quartz	Torr seal epoxy	Gold	Tungsten

Young’s modulus (GPa)	78.31	9.39	70	360
Poisson’s ratio	0.14	0.45	0.44	0.28
mass density (kg·m^−3^)	2651	1600	19300	19300
damping coefficient	1 × 10^−4^	5 × 10^−3^	—	—

### Observation by scanning electron microscope (SEM)

Firstly, in this paper, the whole assembly consisting of tuning fork, tip, and Torr seal epoxy is referred to as the qPlus sensor. The oscillation of the qPlus sensor with a long tilted tip was experimentally observed using a commercial SEM (FEI Quanta FEG 250). The tungsten wire (Alfa Aesar company) used in the experiment had a diameter of 0.025 mm (0.001 in) and a purity of 99.95%. The tip was obtained by AC electrochemical etching in NaOH solution at a concentration of 1 mol·L^−1^. A tungsten wire with a length of 942 μm was attached to the end of the tuning fork with Torr seal epoxy. The angle between the tungsten wire and the prong was 65°. The excitation signal was introduced via mechanical excitation of the base part by a piezo actuator. The excitation amplitude was 1 V. The scanning rate of SEM was 20 μs/pixel. The vibration of the qPlus sensor was observed at room temperature while maintaining a pressure of 10^−3^ Pa.

## Results and Discussion

### Eigenfrequencies and eigenmodes

**Figure 3:**
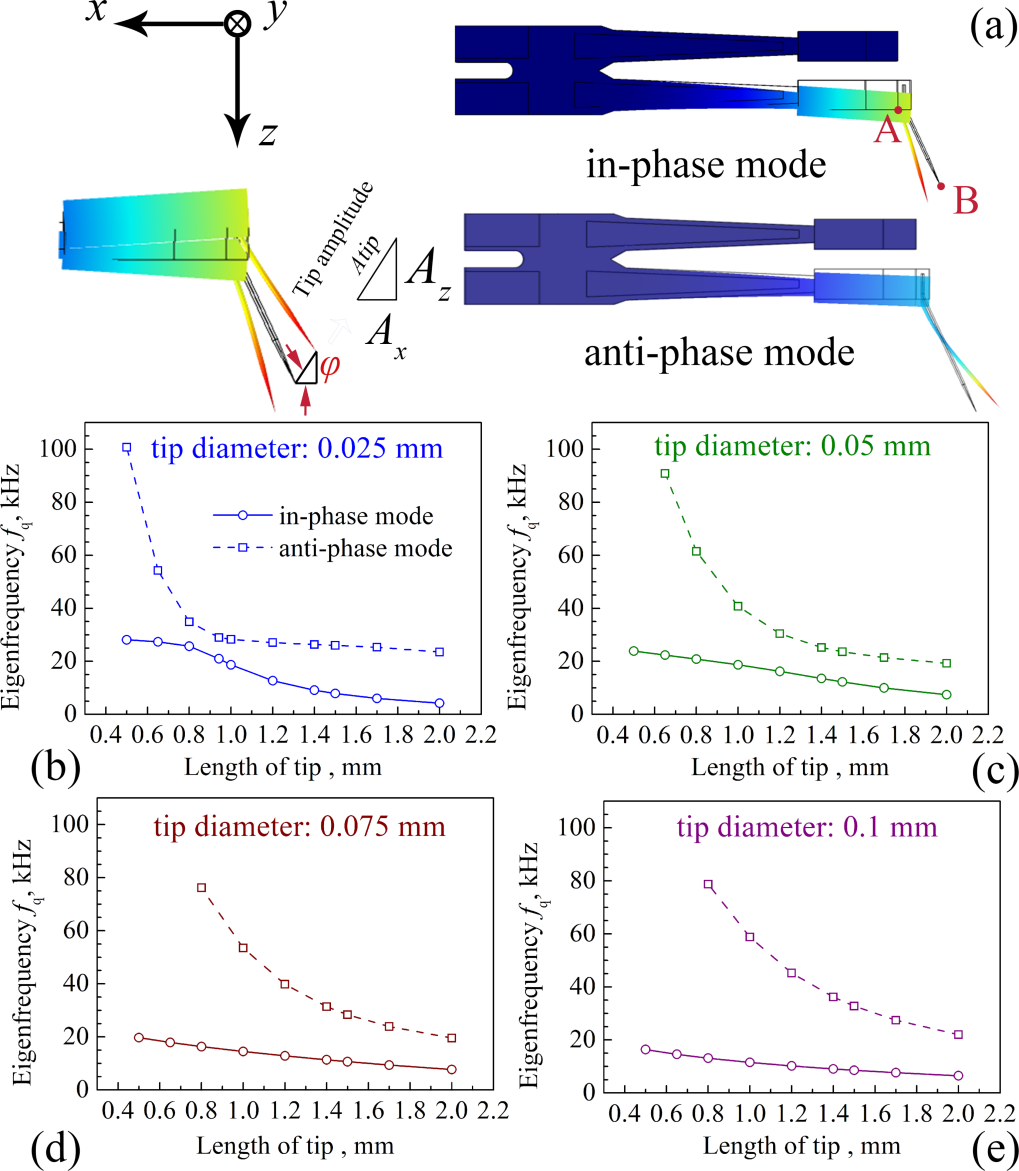
(a) Two different eigenmodes in the simulation. In-phase mode: the tip and the tuning fork deflect in the same direction. Anti-phase mode: the tip and the tuning fork are deflected in the opposite direction. (b–e) The values of *f*_q_ of the four tip diameters with different lengths in both eigenmodes.

The eigenfrequencies of three parts are discussed in this paper. These parts are the qPlus sensor, the prong (the free prong of the tuning fork) with one end fixed and the other end carrying a concentrated load which equals the tip mass, and the independent tungsten tip. These three eigenfrequencies are denoted by *f*_q_, *f*_tf_, and *f*_tip_, respectively. The values of *f*_q_ and *f*_tip_ are obtained by the simulation, and *f*_tf_ is calculated by the method described in [27]. Since the oscillation of the tip is mainly in the tapping mode during scanning, we focus on the modes of the tuning fork prong and the tip oscillating in the *X*–*Z* plane. We found that when the tuning fork oscillates at the first-order eigenfrequency, there are two different vibration eigenmodes of the tip, as shown in Figure 3a. We name the vibration mode in which the tip and the tuning fork are deflected in the same direction the “in-phase” mode. If the tip and the tuning fork are deflected in opposite directions, we name the corresponding vibration mode the “anti-phase” mode. Figure 3b–e summarizes the relations between *f*_q_ and the tip length for the four different tip diameters. In the in-phase mode, the qPlus sensor oscillates at a lower eigenfrequency, and in the anti-phase mode at a higher eigenfrequency. With the increase of tip diameter, the tuning fork shows the vibration of the second-order eigenmode in the anti-phase mode, especially with the shorter tip. This paper does not discuss the second-order eigenmode of the tuning fork, so there are no corresponding frequencies of the anti-phase mode at some tip lengths in Figure 3c–e.

**Figure 4:**
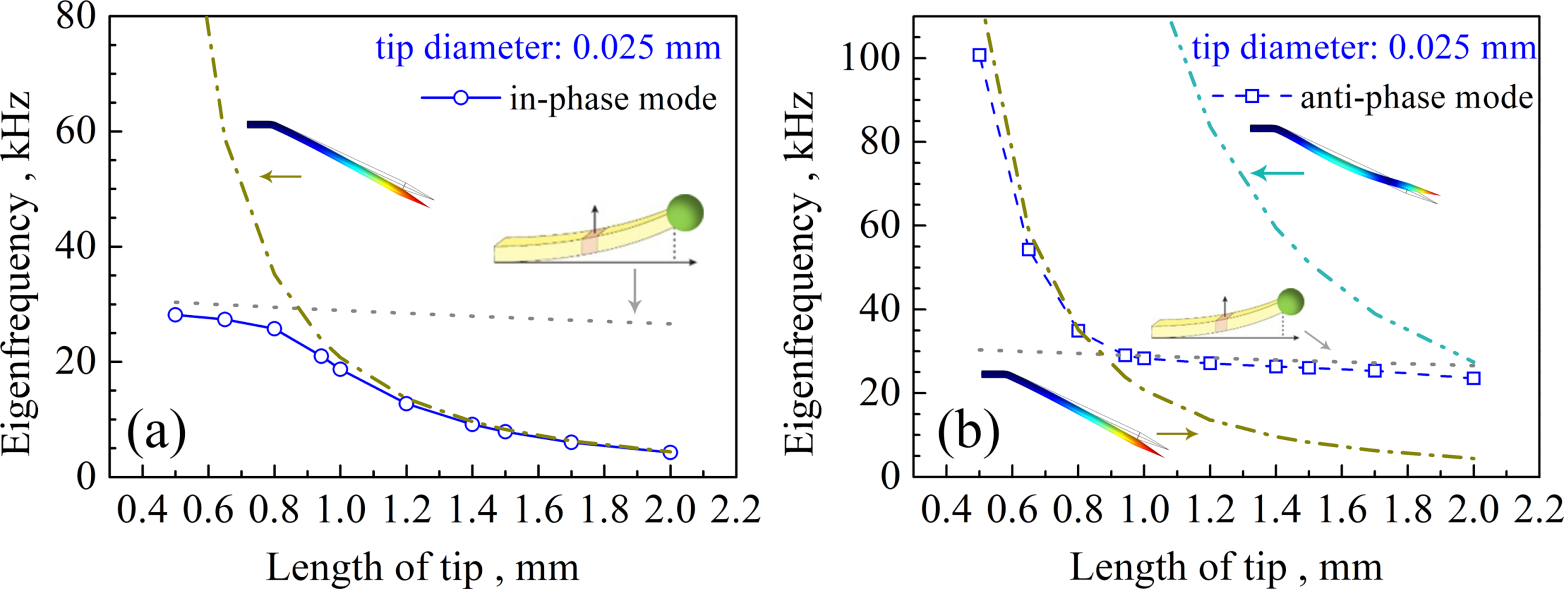
The relations between *f*_q_, *f*_tf_, and *f*_tip_ for the qPlus sensor with a tip diameter of 0.025 mm in the in-phase mode (a) and in the anti-mode (b). The eigenmodes corresponding to *f*_tf_ and *f*_tip_ are shown in the illustrations.

The vibration of the qPlus sensor is the superposition of the oscillation of the tuning fork and the oscillation of the tip. The relations between *f*_q_, *f*_tf_, and *f*_tip_ for the qPlus sensor with a diameter of 0.025 mm tip are shown in Figure 4. The grey dashed lines denote *f*_tf_. When the tip length is 0.5 mm, *f*_q_ of the in-phase mode is very close to the dashed line (Figure 4a), which means that the tuning fork vibrates in resonance. When *f*_q_ moves away from the dashed line and gets closer to *f*_tip_, the vibration of the qPlus sensor is mainly dominated by the tip, while the tuning fork hardly oscillates. The value of *f*_q_ of the anti-phase mode is close to that of *f*_tip_ for a 0.5 mm long tip (Figure 4b), resulting in tip resonance. As the tip length increases, *f*_q_ gradually approaches *f*_tf_, leading to resonance of the tuning fork. If the tip length continues to increase, *f*_q_ will be closer to the second-order *f*_tip_. The superposition vibration characteristics of the qPlus sensor are reflected in the amplitudes of the tuning fork and the tip, which will be discussed later.

### Amplitude of the tuning fork (*A*_tfz_) and its output current

**Figure 5:**
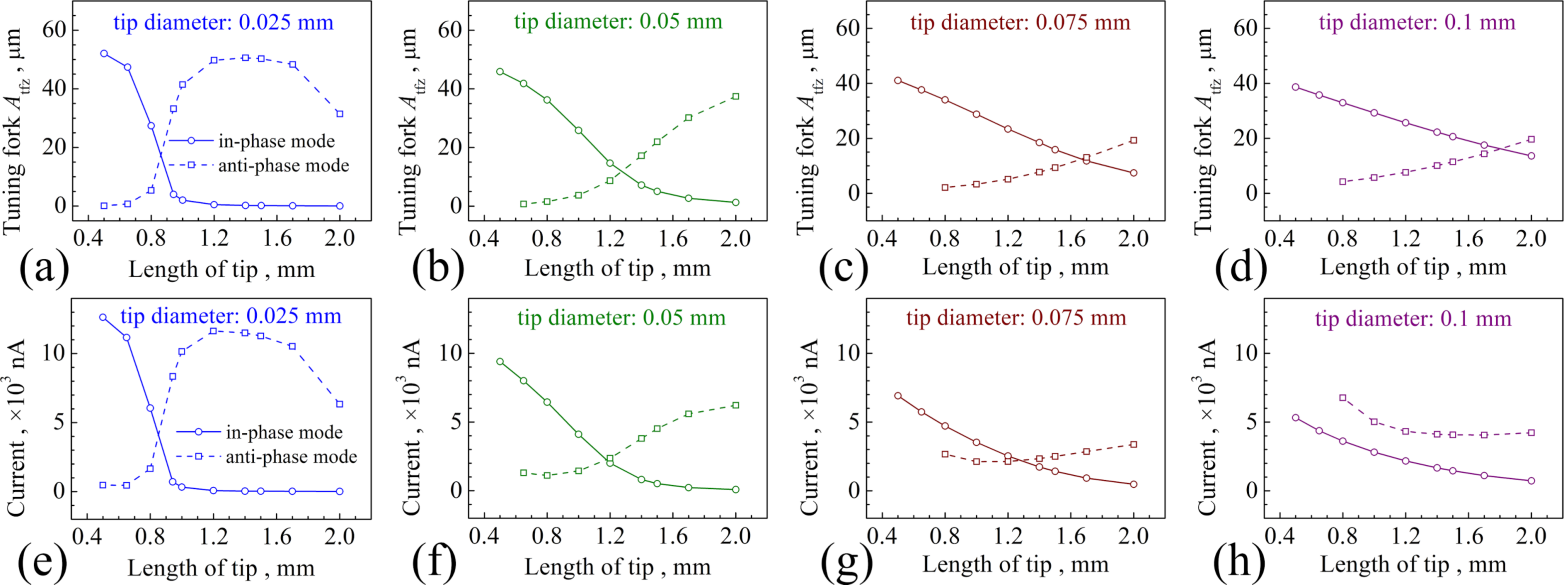
Illustrations of *A*_tfz_ (a–d) and output currents (e–h) as functions of the tip length for the four different tip diameters.

In this paper, the amplitude in the *Z* direction of point A on the tuning fork prong in Figure 3a is denoted as *A*_tfz_. The amplitude of point B at the tip in Figure 3a is denoted as *A*_tip_. *A_z_* and *A_x_* represent the two components of the amplitude of the tip apex in the *Z* and *X* directions, respectively. *A*_tip_ is equal to 
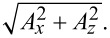
 The angle between *A*_tip_ and *A_x_* is φ. The frequencies near *f*_q_ are scanned by the frequency domain study in COMSOL to obtain the maximum values of *A*_tfz_, *A_x_*, *A_z_*, and the output currents. Figure 5 shows the *A*_tfz_ and the simulated output currents.

Rise and drop of *A*_tfz_ of both modes in Figure 5a–d can be explained by the tuning fork resonance. We take the 0.025 mm tip as an example. When the tip length is 0.5 mm, the tuning fork vibrates resonantly in the in-phase mode (Figure 4a), corresponding to the larger *A*_tfz_ in Figure 5a. The value of *A*_tfz_ decreases due to *f*_q_ moving far away from *f*_tf_. The value of *A*_tfz_ of the anti-phase mode for a 0.5 mm long tip is small because of the large difference between *f*_q_ and *f*_tf_ (Figure 4b). With the increase of the tip length, *f*_q_ gradually gets closer to *f*_tf_, and *A*_tfz_ gradually increases as well. The value of *A*_tfz_ reaches the maximum value when the tip length is 1.4 mm. At larger tip lengths, *f*_q_ gradually moves away from *f*_tf_, while *A*_tfz_ decreases.

Rise and drop of *A*_tfz_ in both modes are also reflected in the simulated output current. Similar to *A*_tfz_, the output current with a shorter tip is larger in the in-phase mode, and the output current with a longer tip is larger in the anti-phase mode (Figure 5e–h). However, we found that the intersection points of the output currents do not coincide with those of *A*_tfz_ (except for the 0.025 mm tip). For example, when the output current generated in the anti-phase mode is larger than that in the in-phase mode (Figure 5g), the value of *A*_tfz_ of the anti-phase mode is lower than that of the in-phase mode (Figure 5c). This is because the frequency of the anti-phase mode is higher, that is, the period is shorter. Although the peak value of the charge *q* of the anti-phase mode, caused by the piezoelectric effect of the tuning fork [1], is smaller than that of the in-phase mode, the maximum value of the partial derivative of *q* with respect to the time (i.e., the peak output current) can be larger than that of the in-phase mode. The result illustrates that the changes of the output current and of *A*_tfz_ with respect to the tip length are not necessarily synchronous.

### Tip oscillation and SEM observation

**Figure 6:**
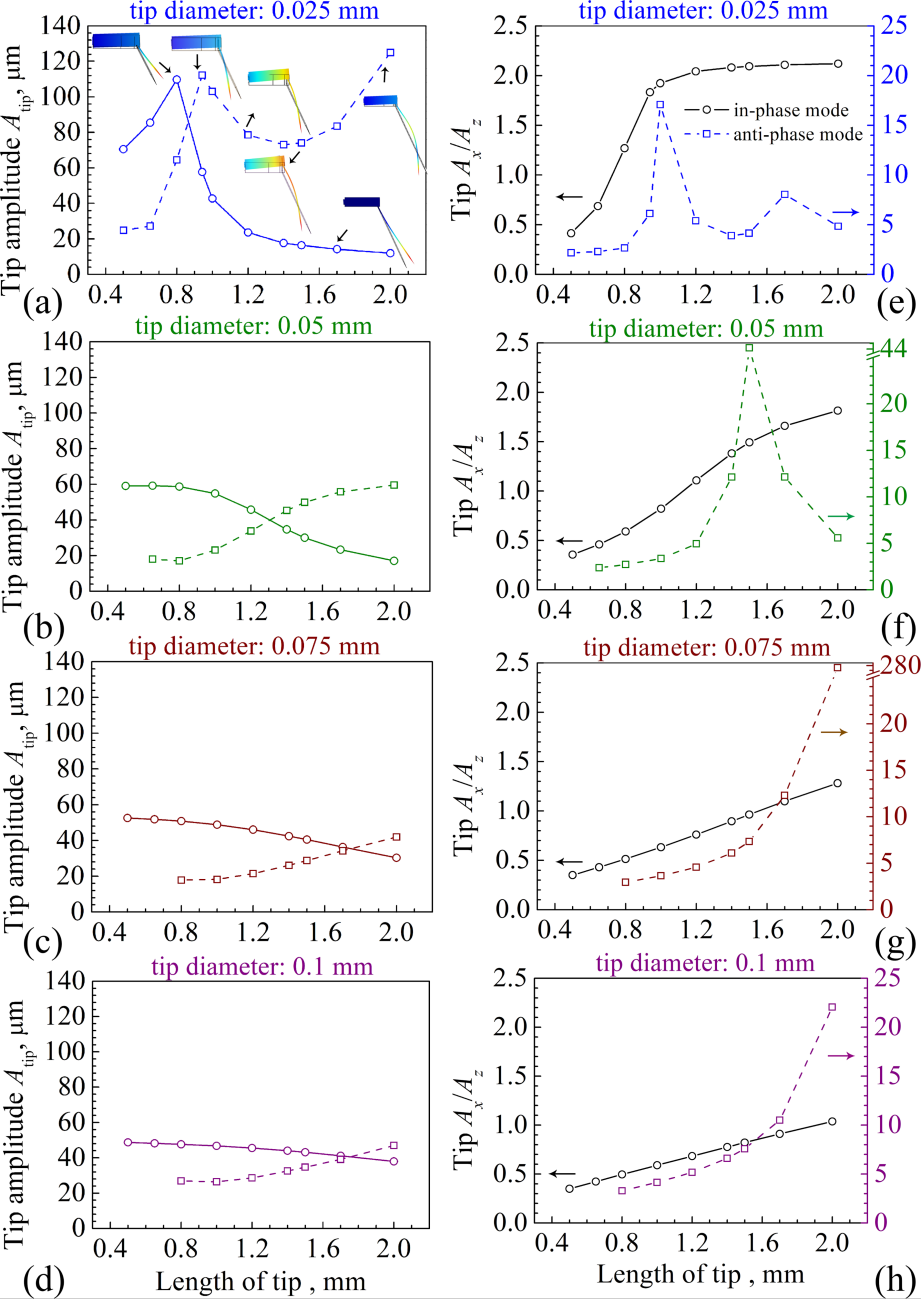
(a–d) *A*_tip_ and (e–h) *A_x_*/*A_z_* as functions of the tip length depicted for the four different tip diameters. The illustrations in (a) show the oscillation behaviors of the 0.025 mm tip with different lengths in both modes.

Since the tip swings around the end of the tuning fork prong, *A*_tip_ is different from *A*_tfz_. We should focus on the vibration of the tip apex itself. Figure 6 gives *A*_tip_ and the ratio between *A_x_* and *A_z_* for the four tip diameters with different lengths. We found that for a fixed tip diameter and tip length, *A*_tip_ is always greater than *A*_tfz_. The value of *A_x_*/*A_z_* is the cotangent value of the angle φ (Figure 3a). If 0 < φ < 45° then *A_x_*/*A_z_* > 1; if 45° < φ < 90° then *A_x_*/*A_z_* < 1. A higher ratio corresponds to a higher proportion of the oscillation parallel to the *X* direction. In contrast, a smaller ratio means the tip oscillation is close to the ideal tapping mode. In the in-phase mode, *A_x_*/*A_z_* for all tip diameters increases with respect to the tip length. However, the increase of *A_x_*/*A_z_* is smaller for 0.075 mm and 0.1 mm tips. When the tip gets thinner, the vibration tends to be parallel to the *X* direction. When the tip gets thicker, the oscillation behaves more like the tapping mode.

The value of *A*_tip_ is determined through the vibrations of the tuning fork prong and the tip. Therefore, the change of *A*_tip_ cannot be simply analyzed by the method for analyzing *A*_tfz_. Again, we take the 0.025 mm tip as an example. The value of *f*_tip_ of the in-phase mode gets closer to *f*_q_ with the increase of the tip length (Figure 4a). Accordingly, the value of *A*_tip_ shows a constant rise when the tip length varies between 0.5 and 0.8 mm (Figure 6a). When the tip length exceeds 0.8 mm, *A*_tfz_ is greatly reduced to almost zero (Figure 5a), which means that the tuning fork hardly oscillates. Therefore, *A*_tip_ is also reduced. In the anti-phase mode, the qPlus sensor vibrates near the first-order *f*_tip_ when the tip length ranges between 0.5 and 0.8 mm (Figure 4b). A longer tip length leads to a larger *A*_tip_ (Figure 6a). At the length of 0.942 mm, the tip apex vibrates almost parallel to the *X* direction. Therefore, *A_x_*/*A_z_* increases greatly (Figure 6e). However, *A*_tip_ begins to decrease when the tip is longer than 0.942 mm. This is caused by the change of *A*_tfz_ and the relative position of the node on the tip. The deflection directions of the tuning fork and the tip are opposite in the anti-phase mode. When the tip is longer than 0.8 mm, *A*_tfz_ increases quickly with respect to the tip length and the node of the tip moves towards the tip apex. Therefore, *A*_tip_ decreases. At a tip length of 1.4 mm, *A*_tip_ reaches its minimal value. Because *f*_q_ of the anti-phase mode gets much closer to the second-order *f*_tip_ when the tip length is longer than 1.4 mm, *A*_tip_ increases at the same time, and the tip apex oscillates parallel to the *X* direction again, resulting in a corresponding increase of *A_x_*/*A_z_*.

**Figure 7:**
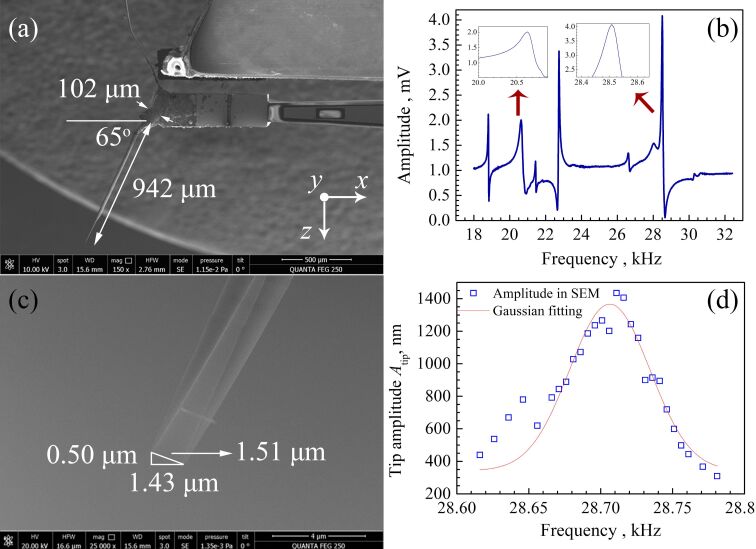
(a) SEM observation of a qPlus sensor with an attached tungsten tip with a length of about 0.942 mm and a diameter of 0.025 mm. (b) Sweeping frequency curve in ambient atmosphere. 20.64 and 28.51 kHz correspond to the values of *f*_q_ of the in-phase mode and the anti-phase mode, respectively, from the simulation. (c) Corresponding SEM images taken around the oscillating tip apex. The value of *A*_tip_ is 1.51 μm, and *A_x_* and *A*_z_ are 1.43 and 0.50 μm, respectively. (d) Blue squares: *A*_tip_ measured from SEM images. Red line: fit with a Gaussian curve. The resonance frequency of 28.71 kHz of the anti-phase mode measured in SEM is slightly higher than that measured in atmosphere due to less air damping.

In order to understand the oscillation behavior of the tip more intuitively, a qPlus sensor with a tip length of 942 μm was fabricated and placed in an SEM to observe the vibration, as shown in Figure 7a. We carried out a series of frequency sweep experiments from 18 to 32 kHz in ambient atmosphere. The obtained response curve is plotted in Figure 7b. We found resonance frequencies of 20.64 and 28.51 kHz in the scanning curve, which are close to the frequencies of 20.98 kHz of the in-phase mode and 28.99 kHz of the anti-phase mode, respectively, from the simulation. When we used a driving frequency between 28.62 and 28.78 kHz in the SEM, the resonance frequency of the qPlus sensor was found at 28.71 kHz (Figure 7d), which is slightly higher than that measured in atmosphere due to less air damping. The oscillation of the apex of the tip is presented in the Figure 7c. The value of *A*_tip_ measured directly from the SEM images was 1.435 μm, *A_z_* and *A_x_* were 0.47 and 1.35 μm, respectively. The ratio between *A_x_* and *A_z_* was 2.85. In the simulation the values were *A_z_* = 18.09 μm, *A_x_* = 110.81 μm*,* and *A_x_*/*A_z_* = 6.12. One possible reason for the lower value of *A_x_*/*A_z_* in the SEM is the expansion of the epoxy glue from the tuning fork to the tip (Figure 7a). This is confirmed by a further simulation (see Supporting Information File 1).

### Tip selection

**Figure 8:**
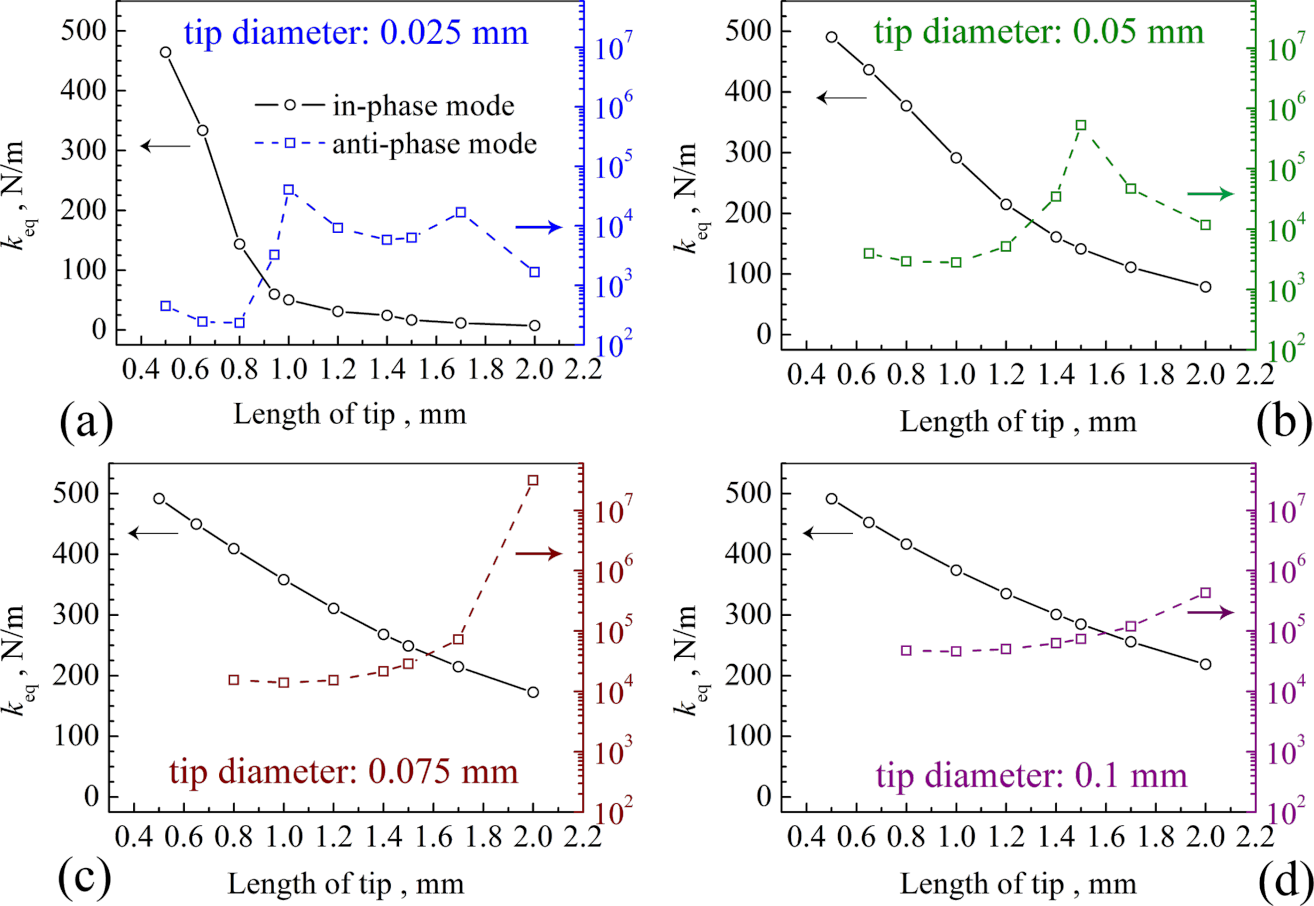
Equivalent stiffness *k*_eq_ of the qPlus sensor as a function of the tip length depicted for the four different tip diameters.

The purpose of optimizing the tip dimensions is to improve the spatial resolution of the measurement. Therefore, we need to consider all physical parameters comprehensively. Firstly, it is necessary to reduce the influence of the long-range force, where *A*_tip_ should be small enough. Secondly, *f*_q_ should be high to minimize frequency noise [1]. However, there is a tradeoff between *A*_tip_ and *f*_q_ in the in-phase mode (Figure 3 and Figure 6). We found a 0.05 mm tip has the best performance when the tip length is 0.65 mm in the anti-phase mode. However, *A_x_*/*A_z_* in the anti-phase mode is 2.36, that is, φ is 23°. In frequency modulation-atomic force microscopy (FM-AFM), the frequency shift Δ*f* of the cantilever is utilized to detect the forces between tip and sample. The shift Δ*f* induced by the vertical force gradient is tan|φ| times as large as that induced by the lateral force gradient [17]. In other words, in the range from 0 to 90°, if φ > 45°, mainly the vertical force gradient contributes to Δ*f*. If φ < 45°, the lateral force gradient has a greater impact. For most cases, it is desirable to detect a larger vertical force gradient signal, so we need *A_x_*/*A_z_* < 1. The in-phase modes were found to fulfil this requirement. Thirdly, we want the stiffness of the qPlus sensor to be large enough to allow for a stable small-amplitude operation [1]. The equivalent stiffness *k*_eq_ of the qPlus sensor is shown in Figure 8, which is calculated from the strain energy and the tip amplitude with an equivalent point-mass model [28]. We see in Figure 8c that for a 0.075 mm tip, there is an optimal tip length of 2 mm that will achieve the maximum *k*_eq_. However, the corresponding vibration is in the anti-phase mode. In the in-phase mode, *k*_eq_ decreases with the increase of the tip length. When the tip length is longer than 0.65 mm, the value of *k*_eq_ of the qPlus sensor with 0.1 mm diameter is larger than that of any tip with another diameter. Thus, 0.1 mm would be the best choice for the tip diameter.

**Figure 9:**
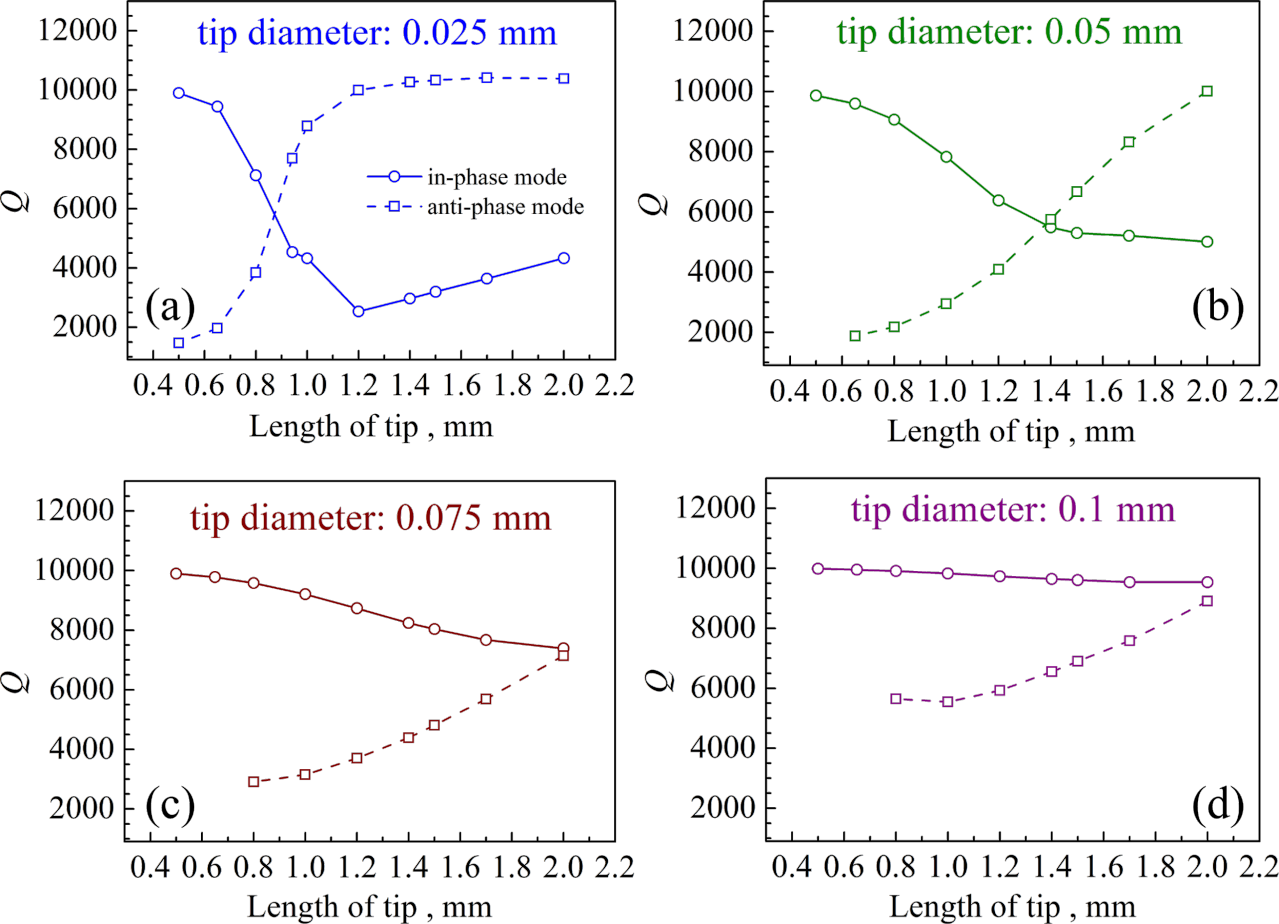
*Q* factor as a function of the tip length depicted for the four different tip diameters.

The last crucial factor to be considered is the *Q* factor. Lower *Q* factors will result in lower stability for both FM-AFM and amplitude modulation AFM (AM-AFM) [17]. As shown in Figure 9, the *Q* factor of the qPlus sensor is higher for shorter tips in the in-phase mode. The *Q* factor of the 0.1 mm tip in the in-phase mode decreases the least when the length increases, and *A*_tip_ is smallest when the tip length is shorter than 1.2 mm. Therefore, in terms of resolution, a 0.1 mm tip with a length less than 1.2 mm in the in-phase mode would exhibit a better performance.

## Conclusion

The oscillation characteristics of qPlus sensors with different dimensions of tungsten tips were studied by using the finite element method. The results reveal that the changes of the output current and the tuning fork amplitude (*A*_tfz_) with respect to the tip length are not necessarily synchronous. The tip amplitude (*A*_tip_) is determined by vibrations of both the tuning fork prong and the tip. A 0.1 mm tip with a tip length smaller than 1.2 mm used in the in-phase mode can improve the spatial resolution. This research provides quantitative data to optimize the tip dimensions and the corresponding vibration modes of a qPlus sensor with a long tilted tip.

## Supporting Information

File 1 Additional simulation results.
