# Supplementary material for: Numerical analysis of vibration modes of a qPlus sensor with a long tip
Source: Beilstein J Nanotechnol. 2021 Jan 21;12:82–92. doi: 10.3762/bjnano.12.7 (PMC7849263; doi:10.3762/bjnano.12.7)
Supplement: File 1 — Additional simulation results. [file Beilstein_J_Nanotechnol-12-82-s001.pdf]

BEILSTEIN JOURNAL OF NANOTECHNOLOGY

Supporting Information

for

Numerical analysis of vibration modes of a qPlus sensor with a long tip

Kebei Chen, Zhenghui Liu, Yuchen Xie, Chunyu Zhang, Gengzhao Xu, Wentao Song and Ke Xu

Beilstein J. Nanotechnol. **2021,** *12,* 82–92. doi:10.3762/bjnano.12.7

Additional simulation results

License and Terms: This is a supporting information file under the terms of the Creative Commons Attribution License (https://creativecommons.org/licenses/by/4.0). Please note that the reuse, redistribution and reproduction in particular requires that the author(s) and source are credited and that individual graphics may be subject to special legal provisions.
The license is subject to the *Beilstein Journal of Nanotechnology* terms and conditions: (https://www.beilstein-journals.org/bjnano/terms)

As we can see from the SEM image in Figure 7a, the epoxy glue on the end of the tuning fork has slightly expanded from the tuning fork to the tip. In this section, we simulated the influence caused by the additional glue volume. A cone of glue around the tip is added to the original glue cuboid (Figure S1). The resonance frequency shifts to 29934 Hz, which is slightly increased. This resonance frequency corresponds to the anti-phase mode, which is strongly influenced by the eigenfrequency of the free-standing tip. For a shorter tip, the resonance frequency is higher. A value of A_x/A_z = 3.86 is obtained from the simulation. This indicates that the glue expansion from the tuning fork to the tip could be the possible reason for the decrease of A_x/A_z.

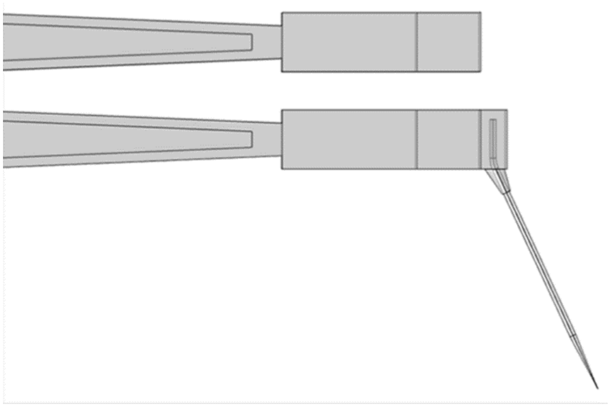

Figure S1: Schematic diagram of the simulation model with an added cone of glue around the tip.
